# The E3 ligase HOIL-1 catalyses ester bond formation between ubiquitin and components of the Myddosome in mammalian cells

**DOI:** 10.1073/pnas.1905873116

**Published:** 2019-06-17

**Authors:** Ian R. Kelsall, Jiazhen Zhang, Axel Knebel, J. Simon C. Arthur, Philip Cohen

**Affiliations:** ^a^Medical Research Council Protein Phosphorylation and Ubiquitylation Unit, School of Life Sciences, University of Dundee, Dundee DD1 5EH, Scotland, United Kingdom;; ^b^Division of Cell Signalling and Immunology, School of Life Sciences, University of Dundee, Dundee DD1 5EH, Scotland, United Kingdom

**Keywords:** LUBAC, Toll-like receptor, TRAF6, IRAK, NEMO

## Abstract

The linear ubiquitin assembly complex (LUBAC) comprises 3 components: HOIP, HOIL-1, and Sharpin, of which HOIP and HOIL-1 are both members of the RBR subfamily of E3 ubiquitin ligases. HOIP catalyses the formation of Met1-linked ubiquitin oligomers (also called linear ubiquitin), but the function of the E3 ligase activity of HOIL-1 is unknown. Here, we report that HOIL-1 is an atypical E3 ligase that forms oxyester bonds between the C terminus of ubiquitin and serine and threonine residues in its substrates. Exploiting the sensitivity of HOIL-1–generated oxyester bonds to cleavage by hydroxylamine, and macrophages from knock-in mice expressing the E3 ligase-inactive HOIL-1[C458S] mutant, we identify IRAK1, IRAK2, and MyD88 as physiological substrates of the HOIL-1 E3 ligase during Toll-like receptor signaling. HOIL-1 is a monoubiquitylating E3 ubiquitin ligase that initiates the de novo synthesis of polyubiquitin chains that are attached to these proteins in macrophages. HOIL-1 also catalyses its own monoubiquitylation in cells and most probably the monoubiquitylation of Sharpin, in which ubiquitin is also attached by an oxyester bond. Our study establishes that oxyester-linked ubiquitylation is used as an intracellular signaling mechanism.

## Significance

The formation of isopeptide bonds between the C-terminal carboxylate of ubiquitin and ε-amino groups of lysine residues on another protein is a major mechanism for regulating protein function. Ubiquitin can also form peptide bonds with the N-terminal α-amino group of another ubiquitin, a reaction catalysed by the HOIP component of the linear ubiquitin assembly complex (LUBAC). Here, we identify the HOIL-1 component of LUBAC as an unusual ligase that catalyses the formation of oxyester bonds between the C-terminal carboxylate of ubiquitin and serine and threonine residues in other proteins. We identify components of the Myddosome as physiological substrates of HOIL-1, indicating a role for HOIL-1 in regulating innate immunity.

Linear ubiquitin assembly complex (LUBAC), a heterotrimeric complex composed of 3 proteins, termed HOIP, HOIL-1, and Sharpin (1–4), is known to catalyze the formation of Met1-linked ubiquitin oligomers (M1-Ub oligomers, also called linear Ub chains) (1). LUBAC is recruited to the signaling complexes that are formed when cells are stimulated with interleukin-1 (IL-1) or ligands that activate Toll-like receptors (TLRs). This triggers the formation of M1-Ub oligomers, which interact with M1-Ub–binding proteins such as the NEMO component of the canonical IκB kinase (IKK) complex (5, 6). The interaction of M1-Ub chains with NEMO facilitates the activation of IKKβ by the protein kinase TAK1 (7, 8), enabling IKKβ to activate key transcription factors that regulate the innate immune system (9). The M1-Ub chains also interact with A20 and A20-binding inhibitor of NF-κB1 (ABIN1), which restrict activation of the TAK1 and IKK complexes and so prevent the overproduction of proinflammatory cytokines and chemokines during TLR signaling that can cause lupus and other autoimmune diseases (reviewed in ref. 10).

The formation of M1-Ub linkages is catalyzed by the HOIP component of LUBAC (1, 7, 11), a member of the “RING-in-between-RING” (RBR) family of E3 ligases. Intriguingly, the HOIL-1 component of LUBAC also possesses the hallmarks of an RBR E3 ligase, but the identity of its physiological substrates and, hence, its function in vivo is unknown. A truncated form of HOIL-1 lacking the Ub-like (UBL) and Npl4 zinc finger (NZF) domains was reported to display no E3 ligase activity, but a near full-length HOIL-1, only lacking the C-terminal 32 amino acid residues, had weak E3 ligase activity (12). A further paper reported that HOIL-1 could undergo autoubiquitylation in vitro (13), while a third reported that HOIP and HOIL-1 were both required for polyubiquitylation of a fragment of NEMO in vitro (14).

Other studies aimed at understanding the physiological roles of HOIL-1 have employed HOIL-1 knockout (KO) mice (15, 16). However, the expression of HOIL-1 is critical for the stability of HOIP in murine cells and vice versa, so that the absence of either protein leads to greatly reduced expression of the other. The embryonic lethality of mice in which HOIL-1 is completely ablated is therefore due to a combination of both HOIL-1 and HOIP deficiency. To investigate the specific role of the E3 ligase activity of HOIL-1 in the innate immune system, we decided to generate a knock-in mouse expressing the E3 ligase-inactive HOIL-1[C458S] protein (12). Here, we report that, in contrast to conventional E3 ligases that catalyze the formation of isopeptide bonds between the C-terminal carboxylate of Ub and ε-amino groups of lysine residues, HOIL-1 catalyses the formation of oxyester bonds between the C-terminal carboxylate of Ub and serine/threonine residues in proteins. We exploit the hydroxylamine sensitivity of these oxyester bonds, and macrophages from knock-in mice expressing the E3 ligase-inactive HOIL-1[C458S] mutant, to identify several of the physiological substrates of HOIL-1 during TLR signaling.

## Results

### HOIL-1–Catalyzed Autoubiquitylation via the Formation of Hydroxylamine-Sensitive Bonds.

To investigate the roles of the E3 ligase activity of HOIL-1, we generated mice with a C458S mutation in the endogenous HOIL-1 gene using CRISPR/Cas9 gene-editing technology. The HOIL-1[C458S] knock-in mice were born at Mendelian frequencies and were of normal size and weight up to 6 months of age (*SI Appendix*, Fig. S1). At this age, they did not show any obvious external abnormality, the spleen and heart were of normal size and weight, and these and other major internal organs did not show any obvious signs of inflammation.

**Fig. 1.**
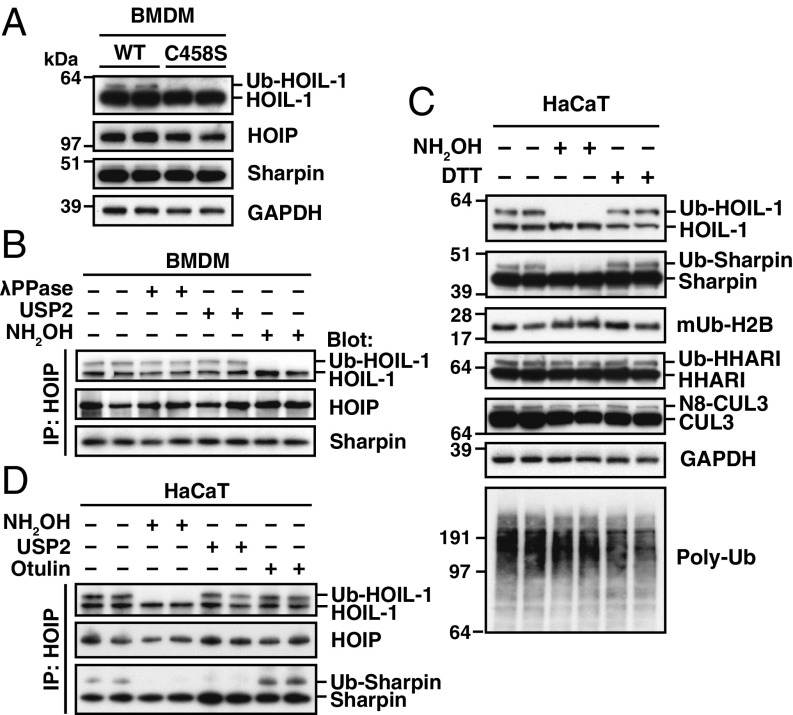
A hydroxylamine-sensitive modification on HOIL-1. (*A*) Immunoblots of BMDM extracts (20 μg of protein) derived from WT and HOIL-1[C458S] knock-in mice. (*B*) LUBAC was immunoprecipitated with anti-HOIP from WT BMDM extracts, then treated with λPPase, USP2, or hydroxylamine, followed by immunoblotting with the antibodies indicated. (*C*) HaCaT cell lysates (30 μg of protein) were treated for 30 min without (−) or with (+) 1.5 M hydroxylamine, subjected to SDS/PAGE without (−) or with (+) 50 mM DTT and immunoblotted with the antibodies indicated. The mUb-H2B antibody detects Histone H2B monoubiquitylated at Lys120. (*D*) LUBAC was immunoprecipitated from HaCaT cell lysates as in *B* and incubated with λPPase in the presence or absence of hydroxylamine, USP2, or Otulin, followed by immunoblotting with the antibodies indicated.

To study the role of HOIL-1 in innate immune signaling, we first examined the expression of HOIL-1, HOIP, and Sharpin in bone marrow-derived macrophages (BMDM) from HOIL-1[C458S] and wild-type (WT) mice and found that they were similar (Fig. 1*A*). This observation indicated that the catalytically inactive HOIL-1 mutant can stabilize HOIP. As observed in other cells (1), HOIL-1 migrated as a doublet in WT BMDM, but the upper band of the doublet was reduced considerably in HOIL-1[C458S] BMDM (Fig. 1*A*). The proportion of the upper band of the doublet was enhanced in HOIL-1 that was coimmunoprecipitated from extracts of WT BMDM by a HOIP antibody (Fig. 1*B*, lanes 1 and 2).

To investigate whether the upper band of the doublet might be a ubiquitylated or phosphorylated form of HOIL-1, we incubated LUBAC immunoprecipitates with Ub-Specific Protease 2 (USP2), a deubiquitylase (DUB) that can hydrolyze all conventional ubiquitin linkage types (17), or with phage λ protein phosphatase (λPPase), but neither treatment converted the upper to the lower band of the doublet (Fig. 1*B*). Recently, MycBP2, a RING-Cys-Relay (RCR) E3 ligase, was reported to catalyze the formation of a hydroxylamine (NH_2_OH)-sensitive oxyester bond between the C-terminal carboxylate of Ub and the amino acids threonine or serine (18). We therefore incubated the LUBAC immunoprecipitates with hydroxylamine and found that the more slowly migrating band of HOIL-1 was converted to the lower band by this treatment (Fig. 1*B*). Similar results were obtained in extracts of HaCaT cells (Fig. 1*C*), a human keratinocyte line that expresses the IL-1 receptor and several TLRs and is useful for studying innate immune signaling. The upper band was unaffected by incubation with dithiothreitol (DTT) (Fig. 1*C*), indicating that it was not formed by attachment to cysteine. Interestingly, Sharpin migrated as a doublet in HaCaT cells (Fig. 1*C*) (but not in BMDM); (Fig. 1*B*), and the upper band of the doublet was also sensitive to hydroxylamine, but not to DTT. This suggested that HOIL-1 may monoubiquitylate Sharpin in HaCaT cells. In contrast to monoubiquitylated HOIL-1, monoubiquitylated Sharpin was hydrolyzed by USP2 as well as hydroxylamine, but neither HOIL-1 nor Sharpin could be deubiquitylated by Otulin (Fig. 1*D*), a DUB reported to hydrolyze M1-Ub linkages specifically (19, 20). Taken together, these observations indicated that Ub was attached to HOIL-1 and Sharpin by an atypical linkage: an oxyester bond. By contrast, the monoubiquitylated forms of histone H2B (21), the RBR E3 ligase HHARI (22), or the neddylated form of CUL3 (23) present in these cell extracts were resistant to hydroxylamine (Fig. 1*C*). In these proteins, the C-terminal carboxylate of Ub or the Ub-like modifier NEDD8 is known to form conventional isopeptide bonds with the ε-amino groups of lysine residues, which are resistant to hydroxylamine. Indeed, none of the major ubiquitylated species detectable in HaCaT cell extracts could be cleaved by hydroxylamine (Fig. 1 *C*, *Bottom*), indicating that the E3 ligase-catalyzed formation of oxyester linkages is a relatively rare event in cells.

**Fig. 2.**
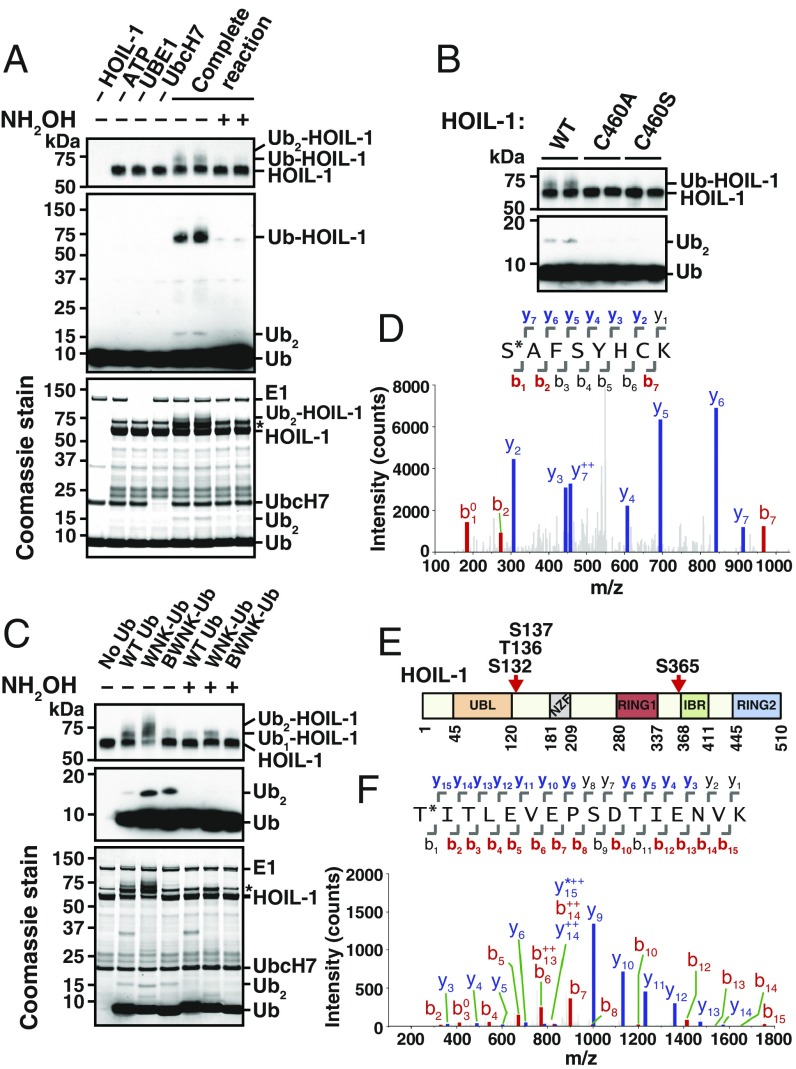
Characterization of the ubiquitin ligase activity of HOIL-1. (*A*) Autoubiquitylation was studied using bacterially expressed His_6_-HOIL-1 and UbcH7. HOIL-1, ATP, UBE1, or UbcH7 were omitted where indicated. The hydroxylamine sensitivity of ubiquitylated HOIL-1 was examined by incubating completed reactions with (+) or without (−) 1.5 M NH_2_OH. The proteins were visualized by immunoblotting with anti-HOIL-1 (*Top*) or anti-ubiquitin (*Middle*) or by staining with Coomassie blue (*Bottom*). The asterisk (*) denotes contaminating bacterial protein in the His_6_-HOIL-1 prep. (*B*) As in *A*, except that reactions contained WT His_6_-HOIL-1 or the indicated His_6_-HOIL-1 mutants. (*C*) As in *A*, except that reactions were carried out using WT Ub, Ub with every lysine mutated to arginine (WNK-Ub), or N-terminally biotinylated WNK-Ub (BWNK-Ub). (*D*) Tandem mass (MS/MS) spectrum of autoubiquitylated HOIL-1 reveals Ser365 as a site of ester-linked ubiquitylation. *y* ions used for scoring are in blue and *b* ions in red. The asterisk (*) on the peptide sequence indicates the site of diglycine attachment. b^0^ on the spectrum plot indicates neutral loss of H_2_O from a *b* ion; y^++^ indicates a doubly charged *y* ion. (*E*) Schematic of HOIL-1 domain architecture with identified sites of Ser/Thr ubiquitylation indicated. IBR, in between RING domain; NZF, NPL4 zinc finger domain; RING, really interesting new gene; UBL, ubiquitin-like domain. (*F*) Annotated tryptic MS/MS spectrum of a HOIL-1–generated ubiquitin dimer reveals Thr12 as a site of ubiquitin ligation. Other details are as in *D*, except that y* on the spectrum plot indicates the neutral loss of NH_3_ from a *y* ion.

To investigate whether HOIL-1 could catalyze the formation of these atypical Ub linkages in vitro, we performed experiments with recombinant human HOIL-1. The purified HOIL-1 was able to catalyze autoubiquitylation, generating predominantly a monoubiquitylated species (Ub-HOIL-1), with lesser amounts of more slowly migrating species, presumably containing more than 1 Ub attached to HOIL-1 (Ub_2_-HOIL-1) (Fig. 2*A*). These autoubiquitylated species were cleaved by incubation with hydroxylamine (Fig. 2*A*), similar to the observations made with the endogenous HOIL-1 present in BMDM and HaCaT cell extracts. These experiments confirmed that HOIL-1 was an E3 ligase likely to catalyze the formation of oxyester bonds.

### HOIL-1–Catalyzed Formation of Ubiquitin Dimers Linked by Hydroxylamine-Sensitive Bonds.

Interestingly, HOIL-1 also catalyzed the formation of hydroxylamine-sensitive ubiquitin dimers (Ub_2_) to a low stoichiometry (Fig. 2 *A*, *Middle* and *Bottom*). Importantly, no Ub_2_ or Ub-HOIL-1 formation took place when HOIL-1 was replaced by HOIL-1[C460A] or HOIL-1[C460S], confirming that these mutants are inactive (Fig. 2*B*). Cys460 of human HOIL-1 is equivalent to Cys458 of mouse HOIL-1. The formation of Ub-HOIL-1 and Ub_2_ was still observed when Ub was replaced by a mutant in which all 7 lysines of Ub were replaced by arginine [with no lysine (WNK)-Ub], and even when the N-terminal α-amino group of WNK-Ub was biotinylated (BWNK-Ub) (Fig. 2*C*). Indeed, the formation of Ub_2_ was more pronounced in these assays, suggesting that these mutant ubiquitins are better substrates for HOIL-1 than unmodified ubiquitin.

### Identification of Ser/Thr Residues in HOIL-1 and Ub That Undergo Ubiquitylation in Vitro.

The ubiquitylated forms of HOIL-1 generated in vitro (Fig. 2*A*) were digested with proteases and analyzed by mass spectrometry. Tryptic cleavage of ubiquitylated proteins generates peptides with characteristic Gly-Gly signatures derived by cleavage of the Arg-Gly-Gly sequence at the C terminus of ubiquitin. These Gly-Gly sequences are normally attached covalently to the ε-amino groups of lysine residues, increasing the molecular mass of such peptides by 114 Da. We detected a peptide with a molecular mass equivalent to the peptide SAFSYHCK plus 114 Da, corresponding to amino acid residues 365–372 of HOIL-1. The fragmentation pattern obtained from the mass spectrum confirmed the peptide’s identity and revealed Ser365 as the only site of ubiquitylation (Fig. 2*D*). Ser365 is located in the RBR domain of HOIL-1 just before the “In Between Ring” (IBR) domain (Fig. 2*E*). The same ubiquitylated peptide was detected in 4 independent experiments. In 1 experiment, we additionally detected peptides with molecular masses equivalent to ^121^QNGDSAYLYLLSAR^134^ and ^135^NTSLNPQELQR^145^ plus 114 Da. The fragmentation patterns obtained from the mass spectra revealed that Ser132, Thr136, and Ser137 were all ubiquitylated (*SI Appendix*, Fig. S2*A*). Overall, peptides corresponding to 80% of the HOIL-1 protein were identified in the mass spectrometry experiments. Therefore, the possibility that additional amino acid residues on HOIL-1 also undergo autoubiquitylation in vitro is not excluded.

When the Ub dimer formed by the action of HOIL-1 was digested with trypsin, we detected a peptide with a molecular mass equivalent to TITLEVEPSDTIENVK plus 114 Da, corresponding to amino acid residues 12–27 of ubiquitin. The fragmentation pattern obtained from the mass spectrum confirmed the peptide’s identity, revealing Thr12 as the site of ubiquitylation (Fig. 2*F*). The same result was observed in 2 independent experiments. In addition, the same peptide with Gly-Gly attached to Thr12 was also identified in the diubiquitylated HOIL-1 formed during the reaction. We detected ubiquitylated forms of Ub[12-27] in 2 other experiments. However, the fragmentation patterns of these peptides indicated that Thr22 and Ser20 were the sites of Ub attachment (*SI Appendix*, Fig. S2*B*). It would therefore appear that in vitro HOIL-1 can ubiquitylate any of 3 Thr/Ser residues within the peptide comprising residues 12–27 of Ub.

### The HOIL-1–Catalyzed Ubiquitylation of IRAK1 and IRAK2 in Macrophages.

Ligands that activate TLRs or the interleukin-1 receptor (IL-1R) induce formation of the Myddosome, a multiprotein complex comprising the liganded receptor, the adaptor protein MyD88, and protein kinases of the IRAK family (24, 25). IRAKs 1 and 2 induce the dimerization and activation of the E3 ligase TRAF6 (26, 27), and IRAK1 phosphorylates and activates the E3 ligases Pellino1 and Pellino2 (28, 29). TRAF6 and Pellino1/2 catalyze the formation of Lys63-linked Ub (K63-Ub) chains that interact with the TAB2 or TAB3 subunits of the TAK1 protein kinase and are required for its sustained activation (30, 31). Some of the K63-Ub chains are attached covalently to IRAK1, IRAK2, and MyD88, and we showed previously that they are ubiquitylated by the HOIP component of LUBAC in IL-1R–expressing HEK293 cells or human THP1 monocytes, generating “hybrid” Ub chains containing both K63-Ub and M1-Ub linkages (32, 33). The M1-Ub oligomers generated during stimulation with TLR ligands can be captured quantitatively on Halo-NEMO beads together with other proteins to which they are attached covalently and noncovalently (32, 33).

**Fig. 3.**
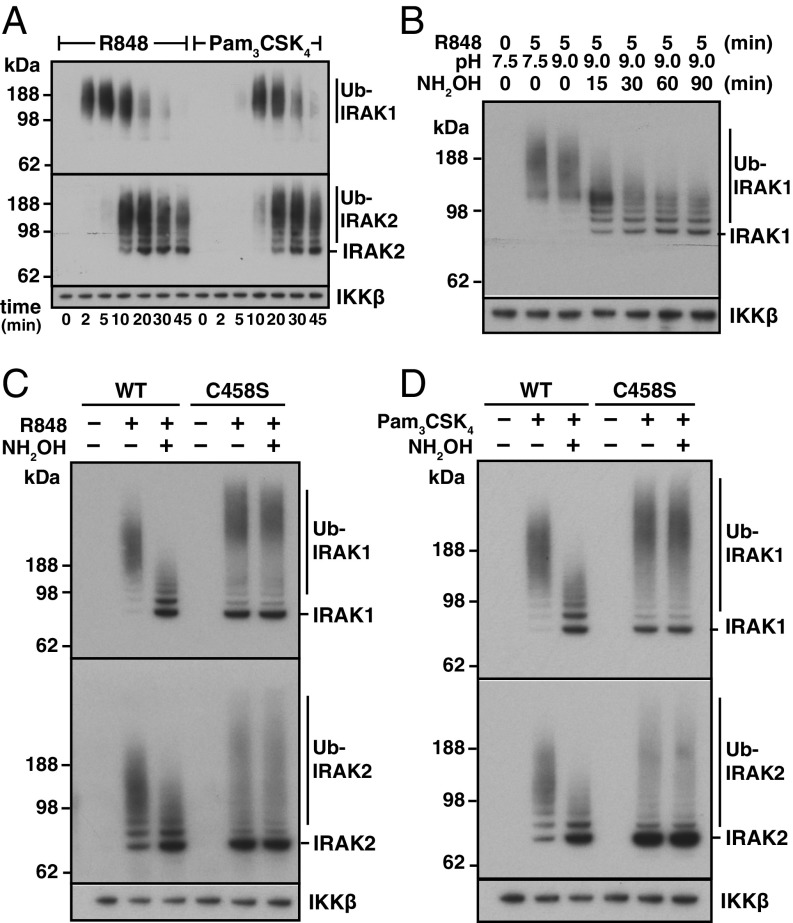
Ubiquitylated IRAK1 and IRAK2 formed during TLR signaling contain HOIL-1–catalyzed, hydroxylamine-cleavable bonds. (*A*) Primary WT BMDM were stimulated with 1 μg/mL R848 or Pam_3_CSK_4_ for the times indicated and lysed. Ubiquitylated proteins were captured on Halo-NEMO beads, incubated for 30 min at 37 °C with λPPase and immunoblotted with antibodies recognizing IRAK1 or IRAK2. IKKβ, which binds to NEMO in a ubiquitin-independent manner, was used as a loading control. (*B*) WT BMDM were stimulated with R848 and ubiquitylated proteins were captured from extracts on Halo-NEMO beads and treated with λPPase as in *A*. Beads were then incubated with 0.5 M hydroxylamine at pH 7.5 or 9.0 for the times indicated. Immunoblotting was as in *A*. (*C* and *D*) BMDM from WT or HOIL-1[C458S] mice were stimulated for 10 min with R848 (*C*) or 20 min with Pam_3_CSK_4_ (*D*) and ubiquitylated proteins captured on Halo-NEMO beads, incubated for 60 min without (−) or with (+) 0.5 M hydroxylamine at pH 9.0 and immunoblotted as in *A*.

In primary BMDM from WT mice, we found that R848 or Pam_3_CSK_4_, which activate the TLR7/8 and TLR1/2 heterodimers, respectively, induced maximal ubiquitylation of IRAK1 at 5–10 min (R848) or 10–20 min (Pam_3_CSK_4_) (Fig. 3 *A*, *Top*), while IRAK2 ubiquitylation became maximal at 10–20 min (R848) or 20–30 min (Pam_3_CSK_4_) (Fig. 3 *A*, *Bottom*). Subsequent experiments were therefore performed after stimulation for 10 min with R848 or 20 min with Pam_3_CSK_4_. To investigate whether the Ub chains attached to IRAK1 contained oxyester bonds, the Halo-NEMO beads were incubated with hydroxylamine. The ubiquitylated IRAK1 was stable for 90 min at 37 °C at pH 7.5 or 9.0 without hydroxylamine, rapidly converted to faster-migrating species when treated with hydroxylamine at pH 9.0 (Fig. 3*B*), but only hydrolyzed slightly at pH 6.8 (*SI Appendix*, Fig. S3). This result is consistent with hydrolysis of an oxyester rather than a thioester bond. Any thioester bonds present should have been hydrolyzed during the prior incubation with λPPase, since DTT was present in the incubations. The size of the Ub chains attached to IRAK1 decreased up to 60 min with no further decrease after 90 min. Further studies were therefore performed after hydroxylamine treatment for 60 min.

To investigate whether the hydroxylamine-sensitive bonds were formed by the action of HOIL-1, we exploited BMDM from HOIL-1[C458S] mice. Following stimulation with R848 (Fig. 3*C*) or Pam_3_CSK_4_ (Fig. 3*D*), we found that the Ub chains attached to IRAK1 and IRAK2 were partially converted to deubiquitylated species in WT BMDM, indicating that the first Ub attached to both proteins was linked via an oxyester bond in some, but not all of the IRAK1/2 molecules. In contrast, although TLR activation still triggered IRAK1 and IRAK2 ubiquitylation in BMDM from HOIL-1[C458S] mice, these Ub chains were completely resistant to hydroxylamine (Fig. 3 *C* and *D*). Taken together, these striking results established that the hydroxylamine-sensitive bonds present in ubiquitylated IRAK1 and IRAK2 are produced by the action of HOIL-1.

The ubiquitin chains attached to IRAK1 and IRAK2 in HOIL-1[C458S] BMDM were much larger than those produced in WT BMDM (Fig. 3 *C* and *D*). This difference was observed at all times examined between 10 and 60 min. The ubiquitylation of IRAK2 occurred more slowly than the ubiquitylation of IRAK1, but was less transient (*SI Appendix*, Fig. S4 *A* and *B*).

**Fig. 4.**
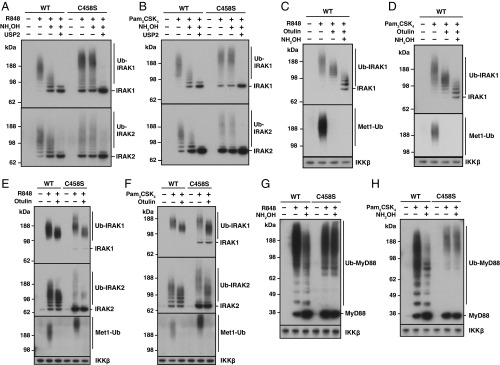
The ubiquitin chains attached to IRAK1, IRAK2, and MyD88 during TLR ligation are initiated by both isopeptide and oxyester bonds. (*A* and *B*) BMDM from WT or HOIL-1[C458S] knock-in mice were stimulated for 10 min with R848 (*A*) or for 20 min with Pam_3_CSK_4_ (*B*) and ubiquitylated proteins captured on Halo-NEMO beads as in Fig. 3 *C* and *D*. Following incubation for 60 min at pH 9.0 without (−) or with (+) 0.5 M hydroxylamine, the beads were incubated for a further 60 min at pH 7.5 without (−) or with (+) 1 μM USP2. Immunoblotting was performed with antibodies recognizing IRAK1 or IRAK2. (*C* and *D*) WT BMDM were stimulated for 10 min with R848 (*C*) or 20 min with Pam_3_CSK_4_ (*D*) and processed as in Fig. 3 *C* and *D*, except that the Halo-NEMO beads were first incubated for 60 min at pH 7.5 without (−) or with (+) 1 μM Otulin and then for 60 min at pH 9.0 without (−) or with (+) 0.5 M hydroxylamine. Immunoblotting was performed with antibodies recognizing IRAK1 or Met1-Ub chains. (*E* and *F*) As in *C* and *D* except that both WT and HOIL-1[C458S] BMDM were used and incubation with hydroxylamine was omitted. (*G* and *H*) As in *C* and *D*, except that immunoblotting was with anti-MyD88.

To investigate why hydroxylamine only induced partial conversion to deubiquitylated IRAK1 and IRAK2, we first incubated the Halo-NEMO beads with hydroxylamine and then with USP2. USP2 hydrolyzed the Ub oligomers that were resistant to hydroxylamine and increased the amount of deubiquitylated IRAK1 and IRAK2 (Fig. 4 *A* and *B*). These experiments indicate that some Ub molecules are attached to IRAK1 and IRAK2 by hydroxylamine-sensitive oxyester bonds, presumably involving serine and/or threonine residues in IRAKs 1 and 2, while others are attached to lysine residues by hydroxylamine-resistant isopeptide bonds. A minor immunoreactive band migrating more slowly than deubiquitylated IRAK1 or IRAK2 was still present after incubation with both hydroxylamine and USP2 (Fig. 4 *A* and *B*). These bands also resisted further incubation with isopeptidases that can cleave other Ub-like molecules from proteins (*SI Appendix*, Fig. S5) and may be a monoubiquitylated species that is resistant to isopeptidases. USP2 also deubiquitylated the hydroxylamine-resistant forms of ubiquitylated-IRAK1/2 generated in BMDM from HOIL-1[C458S] mice (Fig. 4 *A* and *B*), indicating that their ubiquitylation is initiated solely by the formation of isopeptide bonds with ε-amino groups of lysine residues in these proteins.

Incubation with USP2 before hydroxylamine converted the ubiquitylated forms of IRAK1 and IRAK2 to a major deubiquitylated species, and minor monoubiquitylated and diubiquitylated species. The diubiquitylated forms of IRAK1/2, which may be monoubiquitylated at 2 sites, were hydrolyzed by subsequent treatment with hydroxylamine (*SI Appendix*, Fig. S6) showing that, similar to the monoubiquitylated form of HOIL-1 (Fig. 1 *B* and *D*), some oxyester-linked ubiquitins are rather resistant to cleavage by USP2.

Otulin reduced the size of the Ub chains attached to IRAK1 (Fig. 4 *C* and *D*) without generating monoubiquitylated- or diubiquitylated-IRAK1, similar to our earlier findings in human IL-1R and THP1 cells (32, 33). Subsequent treatment with hydroxylamine induced partial conversion to deubiquitylated-IRAK1, indicating that the HOIL-1–catalyzed monoubiquitylation of IRAK1 is followed by elongation with K63-Ub linkages, and then the HOIP-catalyzed formation of M1-Ub linkages attached to preformed K63-Ub oligomers. Otulin also reduced the size of the Ub chains attached to IRAK1 in HOIL-1[C458S] macrophages, which are initiated by isopeptide bond formation (Fig. 4 *E* and *F*), again without generating any monoubiquitylated- or diubiquitylated-IRAK1. Therefore, M1-Ub oligomers are attached to K63-Ub oligomers irrespective of whether the first Ub is linked to IRAK1 by an isopeptide or an oxyester bond(s).

### The HOIL-1 Catalyzed Ubiquitylation of MyD88 in Macrophages.

MyD88 also undergoes ubiquitylation when human cell lines are stimulated with IL-1 or TLR-activating ligands (32). Consistent with these observations, we found that MyD88 became ubiquitylated when WT BMDM were stimulated with R848 (Fig. 4*G*) or Pam_3_CSK_4_ (Fig. 4*H*). MyD88 ubiquitylation took place more slowly than ubiquitylation of IRAK2 (*SI Appendix*, Fig. S4 *C* and *D*). Less ubiquitylated MyD88 was formed when HOIL-1[C458S] BMDM were stimulated with either R848 or Pam_3_CSK_4_, but the Ub chains formed were larger (Fig. 4 *G* and *H* and *SI Appendix*, Fig. S4 *C* and *D*). Most of the ubiquitylated-MyD88 formed in WT BMDM was converted to deubiquitylated MyD88 upon incubation with hydroxylamine and, similar to ubiquitylated IRAK1/2, ubiquitylated-MyD88 formed in HOIL-1[C458S] BMDM was resistant to hydroxylamine (Fig. 4 *G* and *H*). Therefore, most of the Ub chains attached to MyD88 are generated from monoubiquitylated species formed by the action of HOIL-1. Nonubiquitylated MyD88 is captured by Halo-NEMO beads due to its interaction in the Myddosome with ubiquitylated forms of MyD88 and IRAK1/2.

## Discussion

In this paper, we demonstrate that, similar to the RBR ligase HHARI (22, 34), HOIL-1 catalyses the monoubiquitylation of proteins in cells, and we have identified IRAK1, IRAK2, and MyD88 as 3 physiological substrates whose ubiquitylation is stimulated by TLR-activating ligands. HOIL-1 also catalyses its own monoubiquitylation and most probably the monoubiquitylation of Sharpin. In the case of IRAK1, IRAK2, and MyD88, HOIL-1–catalyzed monoubiquitylation is followed by the attachment of additional Ub molecules so that the role of HOIL-1 is to initiate the de novo synthesis of some of the Ub chains that become attached to these proteins during TLR signaling. Importantly, HOIP-generated M1-Ub linkages are also present within these chains, revealing that HOIL-1 and HOIP can ubiquitylate the same proteins in cells. Colocalizing both E3 ligases within LUBAC would therefore appear to permit their corecruitment to the Myddosome, facilitating ubiquitylation of the same substrates in TLR signaling networks.

The most striking observation we made during the present study was that HOIL-1 is an atypical E3 ubiquitin ligase that forms oxyester bonds between the C-terminal carboxylate of Ub and serine and threonine residues in its substrates. We have also established that oxyester-anchored ubiquitin chains are formed in macrophages and that they are catalyzed by HOIL-1. The physiological significance of starting a Ub chain with an oxyester bond has yet to be clarified, but one possibility is that it permits these bonds to be hydrolyzed by a specific DUB. If this were the case, it would permit Ub chains initiated by oxyester bond formation to be detached specifically from their substrates. The possibility that “unanchored” Ub chains have functions distinct from those that remain anchored to other proteins has been discussed by others (35).

We additionally found that HOIL-1 could catalyze the formation of hydroxylamine-sensitive ubiquitin dimers linked via Thr12, Ser20, or Thr22 in vitro (Fig. 2*F* and *SI Appendix*, Fig. S2*B*). Whether these oxyester-linked Ub dimers are formed and present within the hybrid Ub chains produced during TLR signaling has yet to be clarified. However, their presence could explain why the Ub chains attached to IRAK1 and IRAK2 are much smaller in BMDM from WT mice than HOIL-1[C458S] mice (Fig. 3 and *SI Appendix*, Fig. S4). We speculate that oxyester-linked ubiquitins may be a device for capping the further elongation of K63-Ub and M1-Ub oligomers during TLR signaling. Such a mechanism might involve the recruitment of another protein(s) to these unique Ub dimers. The different Ub linkage types present within the hybrid Ub chains attached to IRAK1, IRAK2, and MyD88, and the topology of these chains, is shown schematically in *SI Appendix*, Fig. S7.

In addition to the MycBP2 E3 ligase (18), oxyester-linked ubiquitylation in proteins of the endoplasmic reticulum-associated degradation pathway, catalyzed by certain viral E3 ligases, has been reported (36, 37). Ser/Thr ubiquitylation was inferred in some studies by the observation that ubiquitylation occurred even after every lysine had been mutated to arginine but, in the case of a viral integrase (38) and the cholesterol-regulated degron of squalene monooxygenase (39), was established by mass spectrometry studies. Here, we mapped the sites of Ser/Thr autoubiquitylation in HOIL-1. One site, Ser365, is situated just before the IBR domain, in a region important for Ub binding in other RBR ligase family members (reviewed in ref. 40). It will therefore be interesting to study whether HOIL-1 monoubiquitylation regulates its E3 ligase activity.

The HOIP component of LUBAC not only participates in TLR signaling, but also in signaling by TNF family members, where it has a dual role in activating NF-κB and preventing cell death (reviewed in ref. 41). It also participates in signaling by IL-1 family members, by the peptidoglycan receptors NOD1 and NOD2, and by TLR3, which signals via the adaptor TRIF (19, 42). We therefore anticipate that HOIP and HOIL-1 will be found to ubiquitylate the same substrates in other innate immune signaling networks. There is also evidence that HOIL-1 regulates additional physiological processes where it may operate independently of LUBAC (43). Identifying further substrates of HOIL-1 will undoubtedly be facilitated by exploiting the hydroxylamine sensitivity of oxyester bonds formed by this E3 ligase and further use of the knock-in mice expressing E3 ligase inactive HOIL-1 that were generated in this study.

## Materials and Methods

### Ubiquitylation Assays.

Reactions (20 μL) contained 500 nM His_6_-UBE1, 5 μM UbcH7, 5 μM His_6_-HOIL-1, 50 μM ubiquitin, and 10 mM Mg^2+^-ATP in PBS containing 0.5 mM Tris(2-carboxyethyl)phosphine. Reactions were incubated for 60 min at 37 °C and terminated by the addition of NuPAGE lithium dodecyl sulfate (LDS) gel loading buffer containing 50 mM DTT. Where indicated, reactions were supplemented with 1.5 M hydroxylamine and incubated for a further 60 min at 37 °C before denaturation in LDS. Reaction products were analyzed by protein staining and immunoblotting.

### Affinity Capture of Ubiquitylated Proteins and Treatment with DUBs and NH_2_OH.

To capture M1-Ub and/or K63-Ub chains from cell extracts, and proteins to which they are attached covalently or noncovalently, 2 mg of cell extract protein was incubated for 16 h at 4 °C with Halo-NEMO beads (20 μL of packed bead volume). The beads were washed twice with 1 mL of 10 mM Tris⋅HCl, pH 7.5, 1% (vol/vol) Triton X-100 containing 0.5 M NaCl, and twice with the same buffer without NaCl. The beads were resuspended in 30 μL of 50 mM Hepes pH 7.5, 100 mM NaCl, 2 mM DTT, 0.01% (wt/vol) Brij-35 (protein metallophosphatases [PMP] buffer) containing 1 mM MnCl_2_ and λPPase (100 units per reaction). After incubation for 30 min at 37 °C, beads were washed twice with 1 mL of 10 mM Tris⋅HCl, pH 7.5 containing 1% (vol/vol) Triton X-100, and, where indicated, were resuspended in 30 μL of 19 mM sodium carbonate, 22 mM sodium bicarbonate pH 9.0 without or with 0.5 M hydroxylamine. After incubation for 60 min at 37 °C, the beads were washed twice with 1 mL of 10 mM Tris⋅HCl, pH 7.5 containing 1% (vol/vol) Triton X-100, resuspended in 30 μL of PMP buffer and incubated without or with the deubiquitylases USP2 or Otulin (each at 1 μM). Where indicated, reactions were then incubated for a further 60 min with 0.1 μM vOTU, 1 μM DEN1, or 1 μM SENP1. Proteins were denatured by incubation for 10 min at 37 °C in LDS containing 2.5% (vol/vol) 2-mercaptoethanol, subjected to sodium dodecyl sulfate polyacrylamide gel electrophoresis (SDS/PAGE), transferred to PVDF membranes, and immunoblotted with the appropriate antibodies.

Additional experimental procedures are described in the *SI Appendix*. Experiments on mice were approved by the University of Dundee Ethical Review Board under a UK Home Office project license.

## Supplementary Material

Supplementary File
