# Supplementary material for: The E3 ligase HOIL-1 catalyses ester bond formation between ubiquitin and components of the Myddosome in mammalian cells
Source: Proc Natl Acad Sci U S A. 2019 Jun 17;116(27):13293–8. doi: 10.1073/pnas.1905873116 (PMC6613137; doi:10.1073/pnas.1905873116)
Supplement: Supplementary File [file pnas.1905873116.sapp.pdf]

PNAS
www.pnas.org

Supplementary Information for

The E3 ligase HOIL-1 catalyses ester bond formation between ubiquitin and components of the Myddosome in mammalian cells

Ian R. Kelsall, Jiazhen Zhang, Axel Knebel, J. Simon C. Arthur and Philip Cohen

Corresponding author: Philip Cohen
Email: p.cohen@dundee.ac.uk

This PDF file includes:

Supplementary text
Figs. S1 to S7
References for SI reference citations

www.pnas.org/cgi/doi/10.1073/pnas.1905873116

SI Materials and Methods

General materials. DNA constructs were generated by the DNA cloning team of the MRC Protein Phosphorylation and Ubiquitylation Unit and their DNA sequences verified by MRC-PPU DNA Sequencing and Services (www.dnaseq.co.uk). The plasmids used in this study are available upon request to https://mrcppureagents.dundee.ac.uk. Hydroxylamine (50% wt/vol) and sequencing grade endoproteinase Asp-N were obtained from Sigma-Aldrich. The protein phosphatase from bacteriophage λ (λPPase) was obtained from New England Biolabs. The TLR-activating ligands Pam_3CSK_4 and R848 (Resiquimod) were purchased from InvivoGen. HaloLink Resin and mass spectrometry grade Trypsin Gold from Promega, NuPAGE Bis-Tris gels and NuPAGE LDS sample loading buffer from Thermo Fisher and MOPS and MES gel running buffers from Formedium. Protein G-Sepharose and InstantBlue Coomassie protein stain were obtained from Expedeon, ubiquitin and Mg^{2+}-ATP from Boston Biochem, reagents for cell culture from Gibco and Immobilon-P PVDF membranes from Merck-Millipore. Other solvents and reagents were from Sigma-Aldrich or VWR unless stated otherwise.

Antibodies. Antibodies against HOIP (#SAB2102031) and HOIL-1 (#HPA024185) were purchased from Sigma-Aldrich and anti-Sharpin (#14626-1-AP) from Proteintech. Antibodies that recognize IRAK1 (#4504), GAPDH (#2118), MyD88 (#4283), an antibody that recognises Histone H2B mono-ubiquitylated at Lys120 (#5546) and HRP-coupled secondary antibodies against mouse (#7076) and rabbit (#7074) IgG were obtained from Cell Signaling Technology. HRP-coupled secondary antibody against sheep IgG was from Bio-Rad (#1721017), while HRP-coupled secondary antibody against human IgG was from Southern Biotech (#2040-05). Anti-IRAK2 (#62419) was from Abcam, while anti-IKKβ (#05-535) was from Millipore and anti-ubiquitin (#Z0458) from Dako. The sources of the polyclonal sheep antibodies against HHARI (S622D, fourth bleed), CUL3 (S067D, second bleed) (1), and the HOIP antibody (S174D, fourth bleed) used for immunoprecipitation of the endogenous LUBAC complex (2) have been described previously. The M1-pUb antibody has also been described previously (3).

Generation of HOIL-1[C458S] knock-in mice. A C458S knock-in mutation was created in the *Rbck1* gene encoding HOIL-1 using CRISPR/Cas9-mediated gene-editing technology (performed by Taconic Biosciences GmbH, Köln, Germany). Briefly, a guide RNA was generated to target the sequence AGAAGAAGGACGGCTGTGACTGG in exon 11 of the *Rbck1* gene. *Rbck1* is located on chromosome 2, and prediction of potential off target sites in Chr2 showed that the closest off target site had 4 mismatches including 3 in the seed region; this occurred in 3 places on Chr2, once in the *Rbl1* gene and twice in intragenic regions. Outside Chr2, only two genes had less than 3 mismatches to the oligonucleotide. *Scl38a2* had 2 mismatches, both in the seed region, and *Nptn* had 2, one of which was in the seed region. To generate the knock-in mice, the guide was injected into C57Bl/6NTac zygotes along with Cas9 and an oligonucleotide for homology-directed repair of *Rbck1* exon 11. This oligonucleotide contained a change in codon 458 of *Rbck1* from TGT to AGC, which would change Cys458 to Ser, and also introduce an AlwNI restriction site. Potential founder mice were genotyped for mutation of *Rbck1* by PCR and the presence of the C458S mutation confirmed by sequencing of

PCR products. This generated a *Rbck1* C458S founder that was backcrossed, and transmission of the *Rbck1* C458S allele reconfirmed by PCR genotyping and sequencing. G1 *Rbck1* C458S heterozygous mice were used for further breeding. Genotyping was routinely performed by PCR using the primers CGGCAGACGACAGAGATGC and GGCTGGACTGAGTTCATGGC followed by digestion of the PCR product with Alw N1. For a wild type allele, this results in a band of 509bp while a targeted allele gives bands of 355 and 154bp.

Mice were maintained on a C57BL/6J (Charles River UK) background and provided with free access to food (R&M3 pelleted irradiated diet) and water. Animals were kept in individually ventilated cages at 21°C, 45-65% relative humidity and a 12h/12h light/dark cycle under specific-pathogen–free conditions in accordance with UK and European Union regulations. Experiments on mice were approved by the University of Dundee ethical review board under a UK Home Office project license.

Expression and purification of human proteins. Full length HOIL-1 and mutant proteins with single amino acid substitutions, which were preceded by the sequence MGSSHHHHHHSSGLEVLFQGPGSPEFPGVDSKAAA containing a His_6 tag for affinity purification, were expressed in BL21(DE3) *E. coli* cells. His_6-UbcH7 was expressed in BL21(DE3) *E. coli* cells and His_6-UBE1 was expressed in Sf21 insect cells. After purification by affinity chromatography on Ni-NTA-agarose, the purified proteins were dialysed into Phosphate Buffered Saline (PBS), 1 mM TCEP. The His_6-tag was removed from UbcH7 by cleavage with PreScission Protease and buffer exchanged by size exclusion chromatography into PBS, 1 mM TCEP. The expression and purification of the deubiquitylases USP2-His_6 and GST-Otulin (4), and the isopeptidases vOTU (5), GST-DEN1 (also known as GST-NEDP1) (6) and SENP1 (7) as well as the preparation of Halo-NEMO affinity resin (2) have been described previously. Proteins were stored in aliquots at -80°C.

Cell culture, stimulation and lysis. The human keratinocyte HaCaT cell line was cultured in Dulbecco's Modified Eagle's Medium supplemented with 10% (*v/v*) foetal bovine serum, 2 mM L-glutamine, 100 Units/ml penicillin and 0.1 mg/ml streptomycin. Primary BMDM were obtained by differentiation of bone marrow (extracted from the femur and tibia) using L929 preconditioned medium (8). Where indicated, BMDM were stimulated with 1 µg/ml Pam_3CSK_4 or R848, washed with ice-cold PBS and lysed in 50 mM Tris-HCl pH 7.5, 1 mM EDTA, 1 mM EGTA, 1% (*v/v*) Triton X-100, 270 mM sucrose, 10 mM sodium 2-glycerophosphate, 1 mM sodium orthovanadate, 50 mM sodium fluoride, 5 mM sodium pyrophosphate, 1 mM phenylmethanesulphonyl fluoride, 1 µg/ml aprotinin, 1 µg/ml leupeptin, plus 100 mM iodoacetamide to inactivate deubiquitylases. Cell lysates were clarified by centrifugation (16,000 x g for 10 min at 4°C) and protein concentrations in the supernatants determined by the Bradford method (9).

Immunoblotting and immunoprecipitation. Where indicated, cell lysates were incubated for 30 min at 37°C in the presence of 1.5 M hydroxylamine. NuPAGE LDS sample buffer was added and after incubation for 15 min at ambient temperature, the denatured samples were subjected to SDS-PAGE and immunoblotting. This was

performed using NuPAGE gels and PVDF membranes as described (10). To immunoprecipitate LUBAC, 1 µg of anti-HOIP antibody was incubated for 16 h at 4°C with 1 mg of BMDM extract. 10 µl Protein G-Sepharose (packed bead volume) was added and the samples incubated for a further 60 min at 4°C with end-over-end rotation. The Protein G-Sepharose was collected by brief centrifugation, washed three times with cell lysis buffer containing 200 mM NaCl and once with 50 mM Tris-HCl pH 7.5, 50 mM NaCl and 5 mM DTT. Following the last wash, the Protein G-Sepharose was resuspended in 30 µl of 50 mM HEPES pH 7.5, 100 mM NaCl, 2 mM DTT, 1 mM $MnCl_2$, 0.01% (*w/v*) Brij-35 with or without 100 Units of λPPase, 1 µM USP2, 1 µM Otulin or 1 M hydroxylamine. After incubation for 60 min at 37°C, reactions were terminated by denaturation in NuPAGE LDS sample buffer, the Protein G-Sepharose removed, and the supernatant analysed by SDS-PAGE followed by immunoblotting.

Mass spectrometry. Samples from *in vitro* ubiquitylation reactions were separated by SDS-PAGE and stained with InstantBlue Coomassie stain. Gel slices containing the proteins were excised with a scalpel, alkylated with chloroacetamide (11) and digested with trypsin for 16 h at 37°C (12). In some experiments, a further 3 h incubation with Asp-N proteinase was included to increase the sequence coverage of HOIL-1. Peptides were extracted from the gel slices using 5% formic acid and 50% acetonitrile, followed by concentration in a vacuum centrifuge. They were resuspended in 30µl of 0.1% formic acid (FA) and 10 or 15 µl was injected into the mass spectrometer.

Mass spectrometric analysis was performed by LC-MS/MS on a linear ion trap-orbitrap hybrid mass spectrometer (LTQ-Orbitrap Velos, Thermo Scientific) with an EASY-nESI source (Thermo Scientific) and coupled to a Dionex Ultimate 3000 nano-LC system. The samples were analysed using a 65 min method where the peptides were separated on a PepMap RSLC C18 reverse phase C18 column (2 µm, 100Å, 75 µm × 50 cm) at a flow rate of 300 nl/min and eluted with a 42 min linear gradient from 97% solvent A (0.1% FA in water) to 35% solvent B (80% acetonitrile, 0.08% FA) followed by an increase of solvent B to 99% at 47 min. To identify the most hydrophobic peptides in HOIL-1, the solvent system was changed as follows:- a 30 min linear gradient was used starting at 97% solvent A (0.1% FA in water) and finishing at 100% solvent B (0.1% FA in acetonitrile).

Data was acquired in data dependent mode, switching between MS and MS/MS acquisition automatically. Full scan MS spectra were acquired in the orbitrap over a mass range of 400-1600 m/z with resolution of 60,000 at 400 m/z (after accumulation to an FTMS Full AGC Target: 1,000,000; MSn AGC Target: 50,000). The 20 most intense ions were fragmented by collision induced dissociation with a collision energy of 35eV and analysed in the linear ion trap (Full AGC Target: 30,000. MSn AGC Target: 5,000). Raw files obtained were analysed using Proteome Discoverer 2.0, using Mascot (www.matrixscience.com) as the search engine. The database search parameters were set to detect ubiquitylation of lysine, serine, threonine and tyrosine residues.

Statistics. Pearson's chi-square (χ^2) test (13) was used to compare observed Mendelian frequencies with those theoretical values that might be expected to arise from a random sample, with differences considered significant at $p < 0.05$.

A

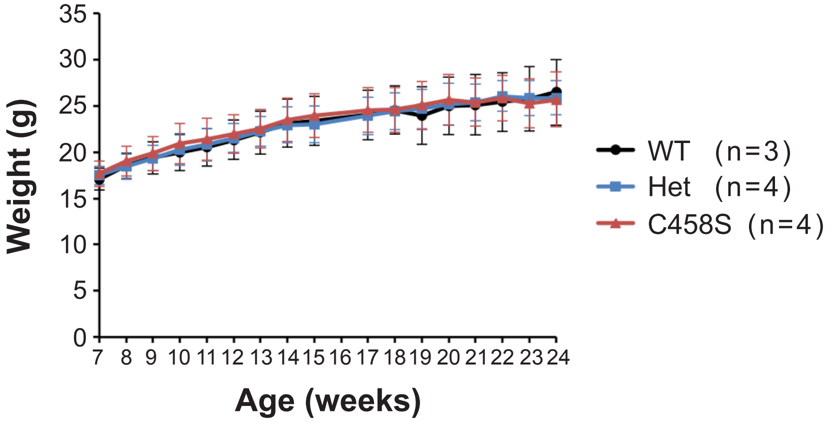

B

	Rbck1$^{WT/WT}$	*Rbck1*$^{WT/C458S}$	*Rbck1*$^{C458S/C458S}$
**Observed number**	27	85	38
**Expected number**	37.5	75	37.5
**Expected ratio**	0.25	0.5	0.25
**Actual ratio**	0.18	0.57	0.25
	**χ^2 = 4.28 *p* = 0.2 *(not significant)***		

Fig. S1. Phenotypic characterisation of HOIL-1[C458S] mice. (*A*) Growth curves of WT, heterozygous and homozygous C458S knock-in mice up to 24 weeks of age are presented as mean body weight ± SEM. n = number of mice in each group (WT = one male, two females, Het = one male, three females, C458S = two males, two females). (*B*) Mouse genotype numbers and ratios obtained from crosses of heterozygous *Rbck1*$^{WT/C458S}$ mice (*Rbck1* is the gene encoding HOIL-1, n=150). Expected genotype distribution is based on Mendelian frequency. The calculated chi-square (χ^2) value indicates no significant deviation from the expected frequencies.

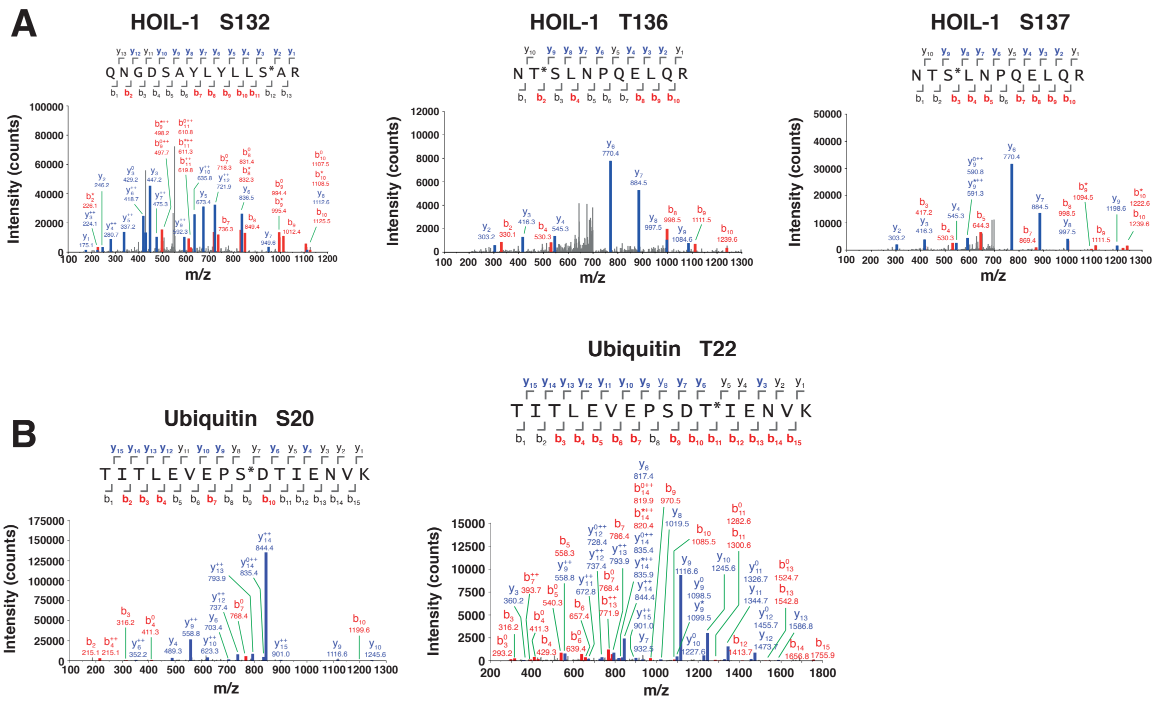

Fig. S2. Additional sites of ester-linked ubiquitylation in HOIL-1 and ubiquitin. (*A*) Tandem mass (MS/MS) spectra of tryptic peptides from auto-ubiquitylated HOIL-1 reveals S132, T136 and S137 as sites of ubiquitin ligation. The asterisk (*) on the upper peptide sequence indicates the site of di-glycine attachment. b^* on the spectrum plot indicates neutral loss of NH_3 from a *b* ion; y^{++} indicates a doubly charged *y* ion; y^0 indicates neutral loss of H_2O from a *y* ion; b^{*++} indicates a doubly charged *b* ion with neutral loss of NH_3; b^{0++} indicates a doubly charged *b* ion with neutral loss of H_2O. (*B*) MS/MS spectra of the HOIL-1-generated ubiquitin dimer reveals S20 and T22 as sites of ester-linked ubiquitylation. Other details are as in A.

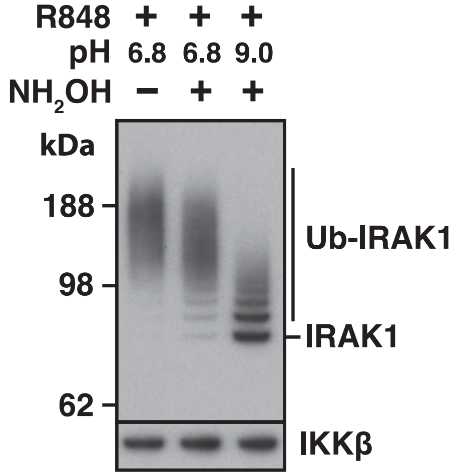

Fig. S3. The hydroxylamine-induced cleavage of ubiquitylated IRAK1 is much slower at pH 6.8 than pH 9.0. WT BMDM were stimulated for 10 min with 1 μg/ml R848 and ubiquitylated proteins captured from cell extracts on Halo-NEMO beads and treated for 30 min at 37°C with λPPase in buffer containing DTT (see Methods). The beads were incubated for 60 min without (−) or with (+) 0.5 M hydroxylamine at pH 6.8 or with 0.5 M hydroxylamine at pH 9.0, then immunoblotted with antibodies recognising IRAK1 or IKKβ. IKKβ, which binds to NEMO in a ubiquitin-independent manner, was used as a loading control. The pH 6.8 buffer was made by adding 1 ml of 15 M hydroxylamine to 29 ml of 100 mM Na_2HPO_4/NaH_2PO_4 (pH 6.0) and adjusting the pH to 6.8 with 0.2 ml of 100% (*v/v*) acetic acid. In the absence of hydroxylamine, the buffer was 100 mM Na_2HPO_4/NaH_2PO_4 pH 6.8. The buffer at pH 9.0 was 19 mM sodium carbonate, 22 mM sodium bicarbonate containing 0.5 M hydroxylamine.

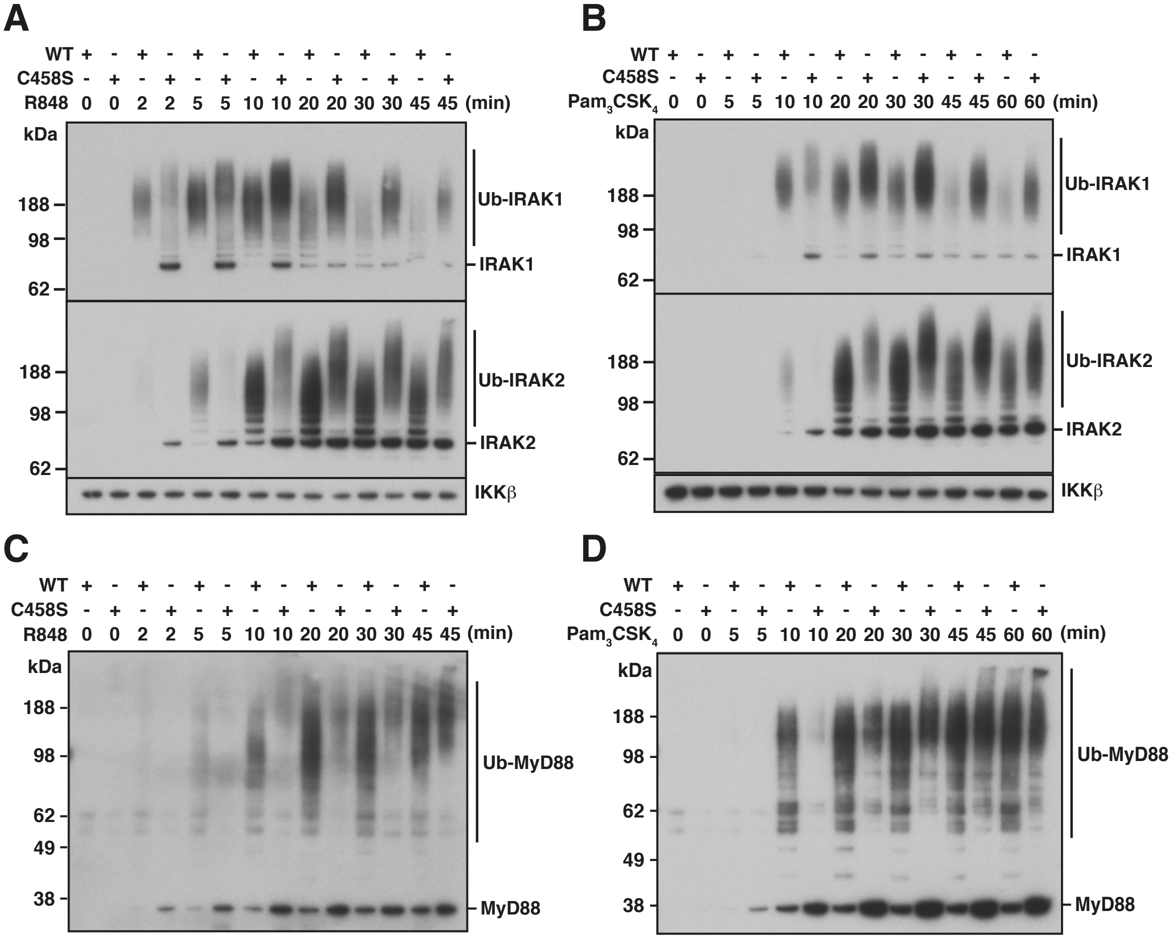

Fig. S4. Rate of formation of ubiquitylated proteins during TLR signalling. BMDM from WT or HOIL-1[C458S] mice were stimulated with 1 μg/ml R848 or 1 μg/ml Pam_3CSK_4 for the times indicated and lysed. Ubiquitylated proteins were captured from the cell extracts on Halo-NEMO beads, incubated for 30 min at 37°C with λPPase, denatured in SDS, subjected to SDS-PAGE and immunoblotted with antibodies recognising IRAK1 or IRAK2 (*A, B*) or MyD88 (*C, D*). IKKβ, which binds to NEMO in a ubiquitin-independent manner, was used as a loading control.

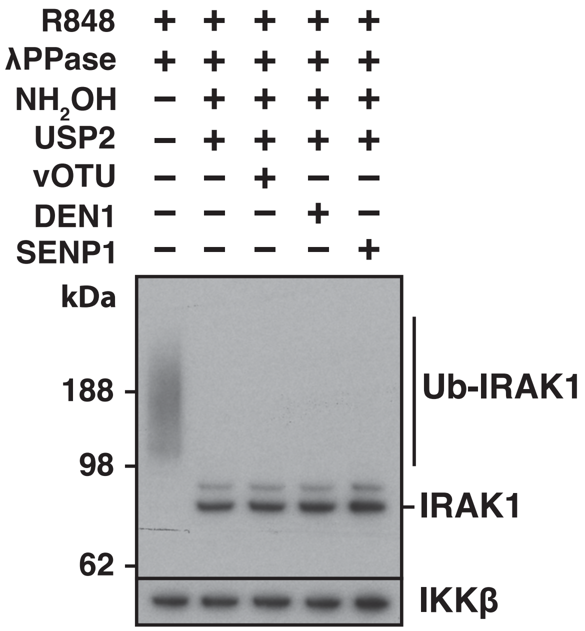

Fig. S5. A more slowly migrating form of IRAK1 produced by incubation with hydroxylamine and USP2 is unaffected by isoproteases that cleave ISG15, NEDD8 and SUMO from proteins. BMDM from WT mice were stimulated for 10 min with R848 (1 µg/ml). Following cell lysis, ubiquitylated proteins were captured on Halo-NEMO beads, treated with λPPase, incubated for 60 min at pH 9.0 without (-) or with (+) 0.5 M hydroxylamine followed by incubation at pH 7.5 with 1 µM USP2. They were then incubated for a further 60 min at pH 7.5 without (-) or with (+) 0.1 µM vOTU (which hydrolyses Interferon-Stimulated Gene 15 from proteins), 1 µM DEN1 (which hydrolyses NEDD8 from proteins) or 1 µM SENP1 (which hydrolyses SUMO from proteins) at pH 7.5, and denatured in SDS. Following SDS-PAGE the captured proteins were immunoblotted with antibodies recognising IRAK1 or IKKβ as a loading control.

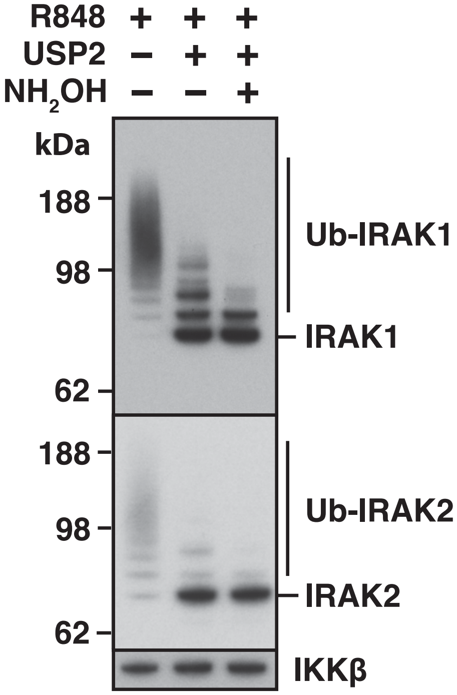

Fig S6. The oxyester-linked ubiquitin attached directly to IRAK1 and IRAK2 is resistant to USP2. WT BMDM were stimulated for 10 min with 1 μg/ml R848, the ubiquitylated proteins captured on Halo-NEMO beads and treated for 30 min at 37°C with λPPase. Following incubation for 60 min at pH 7.5 without (−) or with (+) 1 μM USP2, the beads were washed and then incubated for a further 60 min at pH 9.0 with 0.5 M hydroxylamine. Following SDS-PAGE and transfer to PVDF membranes, captured proteins were immunoblotted with antibodies recognising IRAK1, IRAK2 and IKKβ (loading control).

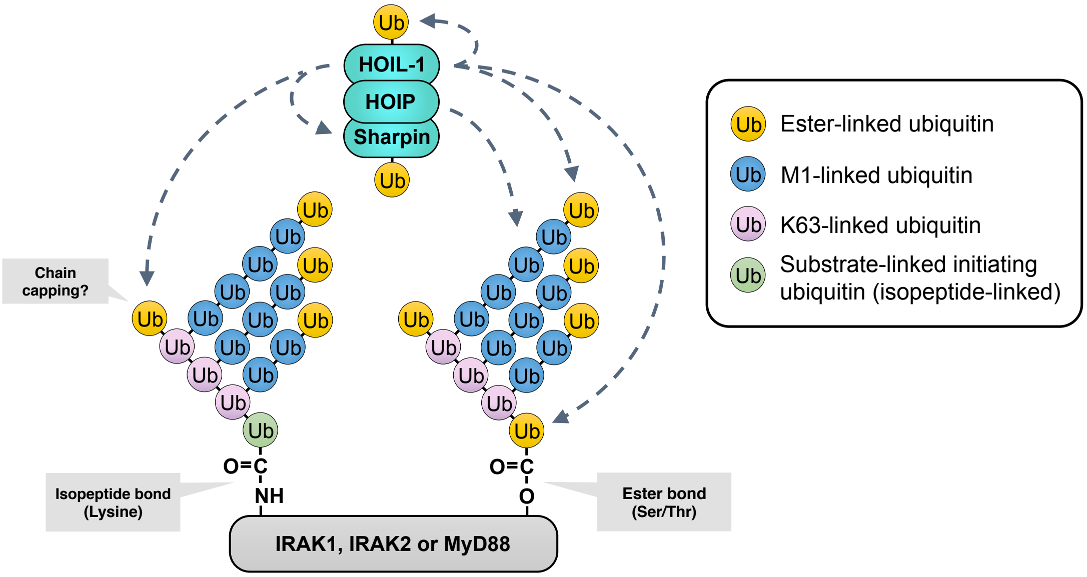

Figure S7. Schematic of the linkage types present within the ubiquitin chains attached to IRAK1, IRAK2 and MyD88. Earlier publications established the presence of hybrid ubiquitin chains containing both K63-Ub and M1-Ub linkages. The present study establishes that two types of Ub chain are attached to IRAK1, IRAK2 and MyD88. One is initiated by the attachment of ubiquitin to a serine or threonine residue on these proteins and is catalysed by HOIL-1, while the other is initiated by the formation of an isopeptide bond to a lysine residue. Ubiquitin is also attached covalently to HOIL-1 and Sharpin by oxyester bonds. HOIL-1 can also catalyse the formation of ubiquitin dimers linked by an oxyester bond *in vitro*, but whether these linkages are present in the ubiquitin chains that become attached to IRAK1, IRAK2 and MyD88 during TLR signalling is not established. In the schematic we speculatively place such linkages at the terminal K63-Ub and M1-Ub linkages as a "capping" mechanism to explain why the ubiquitin chains that become attached to IRAK1 and IRAK2 are much larger in macrophages from mice expressing an E3 ligase-inactive mutant of HOIL-1.

References

1. Kelsall IR*, et al.* (2013) TRIAD1 and HHARI bind to and are activated by distinct neddylated Cullin-RING ligase complexes. *EMBO J* 32:2848-2860.
2. Emmerich CH*, et al.* (2013) Activation of the canonical IKK complex by K63/M1-linked hybrid ubiquitin chains. *Proc Natl Acad Sci U S A* 110:15247-15252.
3. Matsumoto ML*, et al.* (2012) Engineering and structural characterization of a linear polyubiquitin-specific antibody. *J Mol Biol* 418:134-144.
4. Emmerich CH*, et al.* (2016) Lys63/Met1-hybrid ubiquitin chains are commonly formed during the activation of innate immune signalling. *Biochem Biophys Res Commun* 474:452-461.
5. Ritorto MS*, et al.* (2014) Screening of DUB activity and specificity by MALDI-TOF mass spectrometry. *Nat Commun* 5:4763.
6. Kelsall IR*, et al.* (2019) Coupled monoubiquitylation of the co-E3 ligase DCNL1 by Ariadne-RBR E3 ubiquitin ligases promotes cullin-RING ligase complex remodeling. *J Biol Chem* 294:2651-2664.
7. Shen LN, Dong C, Liu H, Naismith JH, & Hay RT (2006) The structure of SENP1-SUMO-2 complex suggests a structural basis for discrimination between SUMO paralogues during processing. *Biochem J* 397:279-288.
8. Pauls E*, et al.* (2013) Two phases of inflammatory mediator production defined by the study of IRAK2 and IRAK1 knock-in mice. *J Immunol* 191:2717-2730.
9. Bradford MM (1976) A rapid and sensitive method for the quantitation of microgram quantities of protein utilizing the principle of protein-dye binding. *Anal Biochem* 72:248-254.
10. Emmerich CH & Cohen P (2015) Optimising methods for the preservation, capture and identification of ubiquitin chains and ubiquitylated proteins by immunoblotting. *Biochem Biophys Res Commun* 466:1-14.
11. Nielsen ML*, et al.* (2008) Iodoacetamide-induced artifact mimics ubiquitination in mass spectrometry. *Nat Methods* 5:459-460.
12. Shevchenko A, Tomas H, Havlis J, Olsen JV, & Mann M (2006) In-gel digestion for mass spectrometric characterization of proteins and proteomes. *Nat Protoc* 1:2856-2860.
13. Pearson K (1900) On the criterion that a given system of deviations from the probable in the case of a correlated system of variables is such that it can be reasonably supposed to have arisen from random sampling. *Lond Edinb Dubl Phil Mag* 50:157-175.
